# Functional Recovery following Repair of Long Nerve Gaps in Senior Patient 2.6 Years Posttrauma

**DOI:** 10.1097/GOX.0000000000003831

**Published:** 2021-09-22

**Authors:** Christian A. Foy, William F. Micheo, Damien P. Kuffler

**Affiliations:** From the * Section of Orthopedic Surgery, Medical Sciences Campus, University of Puerto Rico, San Juan, Puerto Rico; † Department of Physical Medicine & Rehabilitation, Medical Sciences Campus, University of Puerto Rico, San Juan, Puerto Rico; ‡ Institute of Neurobiology, Medical Sciences Campus, University of Puerto Rico, San Juan, Puerto Rico.

## Abstract

Sensory nerve grafts are the clinical “gold standard” for repairing peripheral nerve gaps. However, reliable good-to-excellent recovery develops only for gaps less than 3–5 cm, repairs performed less than 3–5 months posttrauma, and patients aged less than 20–25 years. As the value of any variable increases, the extent of recovery decreases precipitously, and if the values of any two or all increase, there is little to no recovery. One 9-cm-long and two 11-cm-long nerve gaps in a 56-year-old patient were repaired 2.6 years posttrauma. They were bridged with two sensory nerve grafts within an autologous platelet-rich plasma-filled collagen tube. Both were connected to the proximal ulnar nerve stump, with one graft end to the distal motor and the other to the sensory nerve branches. Although presurgery the patient suffered chronic level 10 excruciating neuropathic pain, it was reduced to 6 within 2 months, and did not increase for more than 2 years. Motor axons regenerated across the 9-cm gap and innervated the appropriate two measured muscles, with limited muscle fiber recruitment. Sensory axons regenerated across both 11-cm gaps and restored normal topographically correct sensitivity to stimuli of all sensory modalities, including static two-point discrimination of 5 mm, and pressure of 2.83 g to all regions innervated by both sensory nerves. This novel technique induced a significant long-term reduction in chronic excruciating neuropathic pain while promoting muscle reinnervation and complete sensory recovery, despite the values of all three variables that reduce or prevent axon regeneration and recovery being simultaneously large.

## INTRODUCTION

The capacity of sensory nerve grafts, the clinical “gold standard” technique, to restore function to peripheral nerves with a gap^1,2^ is significantly limited by increasing gap length, time between trauma and repair, and patient age. Thus, reliable good-to-excellent recovery develops only across gaps less than 3–5 cm,^2–7^ repairs performed less than 3–5 months posttrauma,^4,5^ and patients aged less than 20–25 years.^3–5^ As the value of any variable increases, the extent of recovery decreases.^3–19^ As any two variables increase, there is a precipitous decrease in recovery.^20^ If all three increase simultaneously, there is very limited to no recovery.^4,9,20,21^

## MATERIALS AND METHODS

### Patient

Here, we report on a 56-year-old man who presented 2.6 years after a zone 4–5 ulnar nerve laceration resulting in severe intrinsic muscle atrophy and loss of sensation to ring and small finger digital ulnar nerve distribution.

### Surgery

**Fig. 1.**
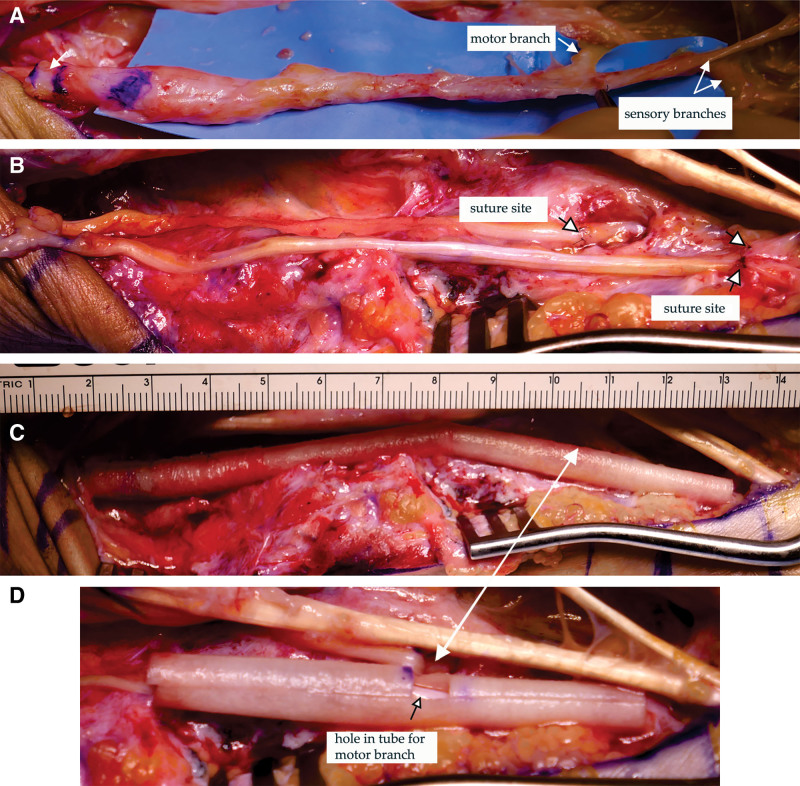
Technique for reparing three long nerve gaps. Repair of one 9-cm-long and two 11-cm-long ulnar nerve gaps. A, Exposed ulnar nerve in the wrist/palm showing the severely damaged nerve. The arrow near the blue lines marks where the nerve will be cut. The other arrows indicate where the motor and two sensory branches will be cut. B, Two sural nerve grafts with their distal ends secured to the motor and two sensory nerve branches (marked by arrows). The proximal ends of the grafts are not yet secured to the proximal ulnar nerve stump. C, Completed nerve gap repair after injecting the PRP inside the collagen tube. D, Blow-up of the distal collagen tube with a hole cut into one side, marked by a black arrow, through which the motor branch entered into the collagen tube. The white arrow between panels C and D indicates the location of the hole in the completed collagen tube.

The nerve trauma region was exposed from the wrist to the trifurcation of its first motor and two sensory branches (Fig. 1A). The nerve stumps were trimmed to remove visible damaged tissue.

A 22-cm length of sural nerve was removed and cut into two lengths. The ends of both grafts were loosely secured to the proximal ulnar nerve stump, while the other end of one was loosely secured to the distal motor nerve branch and the other to the two distal sensory nerve branches (Fig. 1B).

The nerve grafts were surrounded with a tube of two 5-cm and one 3-cm lengths of NeuroMend collagen tubes (Collagen Matrix Inc., Oakland, N.J.) (Fig. 1C). The tubes have a longitudinal slit and are self-closing with a 25% overlap, which allows opening the tubes and slipping the nerve grafts inside.

Because the distal motor nerve branch was proximal to the distal end of the tube, a hole was cut in its side into which the motor branch was slipped (Fig. 1C, D). The collagen tubes were adjusted to ensure overlapping ends and that the anastomosis sites were 2 mm or more inside the collagen tube.

### Platelet-rich Fibrin

An estimated 6 cc of platelet-rich plasma (PRP) was prepared and injected as previously described.^22^

## RESULTS

After removing the damaged nerve tissue, the motor branch had a 9-cm gap, and the two sensory nerve branches had 11-cm gaps. Electrodiagnostic and physical examinations performed 1.75 years postrepair established the presence of ulnar motor axon electrical continuity across the 9-cm gap to the abductor digit minima and adductor pollicis muscles. Evoked motor axon action potentials to the adductor digiti minimi of the little finger had a prolonged latency of 4.13 versus 2.90 ms for the opposite hand and a decreased amplitude of 0.023 versus 10.1 mV, whereas those to the first dorsal interosseous had an adequate latency of 3.49 versus 3.90 ms, and a decreased amplitude of 1.07 versus 9.24 mV.

Electromyography studies established motor axon reinnervation of adductor digiti minimi and first dorsal interosseous muscle fibers. Evoked contractions were minimal due to poor muscle fiber recruitment.

Nerve conduction studies established electrical continuity across both 11-cm sensory nerve gaps, with a prolonged action potential latency of 4.50 versus 3.45 ms for the opposite hand and a decreased amplitude of 3.1 versus 9.5 mV. The dorsal ulnar sensory nerve branch had normal latency parameters of 2.95 versus 2.50 ms and 12.6 versus 7.90 mV amplitudes.

Sensory tests showed recovery of normal levels of topographically correct sensitivity to stimuli of all sensory modalities to all appropriate areas of the skin of both the small and ring fingers. This included sensitivity to light stroking, pinprick, deep pressure, hot and cold, and vibration, and correct proprioception to MCP, PIP, and DIP movements. The little finger developed static two-point discrimination of 5 mm, and the ring finger of 6–8 mm. Both fingers had a pressure sensitivity of 2.83 g. The sensory recovery by both sensory nerve branches was S4.

Although presurgery the subject suffered level 10 chronic excruciating neuropathic pain, it was reduced to 6 within 2 months. Subsequently, the level did not change for more than 2 years.

## DISCUSSION

Although sensory nerve grafts are the clinical “gold standard” technique for repairing nerve gaps,^1,2^ recovery is often disappointing.^17,23,24^ This is attributed to the negative influence of the increasing values of gap length, time between trauma and repair, and patient age.^12^ Thus, reliable good-to-excellent recovery is only across gaps less than 3–5 cm,^2–7^ repairs performed less than 3–5 months posttrauma,^4,5^ and patients aged less than 20–25 years.^3–5,19,25,26^ As any value increases, recovery decreases precipitously.^18^ When all three values increase simultaneously, there is very limited to no recovery.^4,9,20,21^

Three long nerve gaps of a 56-year-old patient were repaired 2.6 years posttrauma using sensory nerve grafts within a PRP-filled collagen tube. Despite a 9-cm motor nerve gap, muscle fibers of the appropriate muscles were reinnervated. Limited contractions are attributed to poor muscle fiber recruitment due to massive muscle fiber atrophy.

Sensory axons regenerated entirely across both 11-cm gaps and established normal levels of topographically correct sensitivity to stimuli of all sensory modalities by all finger regions normally innervated by the two nerves. This included a static two-point discrimination of 5 mm and 6–8 mm, and a pressure sensitivity of 2.83 gm, comparable to the contralateral hand.

Although the patient suffered excruciating level 10 chronic neuropathic pain before surgery, it was reduced to 6 within 2 months. Subsequently, the pain did not change for more than 2 years.

When the values of all three variables that negatively influence axon regeneration are simultaneously large, sensory nerve grafts do not induce the observed axon regeneration, reinnervation, and recovery. This suggests that platelet-released factors promoted the recovery. This hypothesis is consistent with animal^27–40^ and clinical^22,41,42^ studies showing that PRP enhances axon regeneration.

In conclusion, despite simultaneous long nerve gaps, long repair delay, and older age, bridging nerve gaps with a sensory nerve graft within a PRP-filled collagen tube significantly reduces chronic neuropathic pain while inducing axon regeneration and recovery under conditions where sensory nerve grafts alone are not effective. The use of PRP in this study is off-label.

## ACKNOWLEDGMENT

This study was performed under a local IRB-approved clinical study protocol and conformed to the Declaration of Helsinki.
